# “I want to kill my mother”

**DOI:** 10.1192/j.eurpsy.2022.1800

**Published:** 2022-09-01

**Authors:** H.T. Tan

**Affiliations:** Institute of Mental Health, Psychiatry, Singapore, Singapore

**Keywords:** Anti-NDMA receptor encephalitis, paediatric auto immune encephalitis, homicidal ideations

## Abstract

**Introduction:**

The incidence of Anti-NMDA receptor encephalitis is 1.5 per million population per year but it has only been described sporadically in the paediatric population. The median age of onset is 21 years old. In children, seizures, abnormal movements, insomnia, and irritability are more frequently identified than psychosis or abnormal behavior. Early detection and treatment can improve treatment outcomes.

**Objectives:**

Presentation of a case of a paediatric patient with Anti-NMDA receptor encephalitis displaying homicidal behavior towards her family members

**Methods:**

Case report

**Results:**

A 12 year old with no significant past psychiatric and medical history, was brought in by police to the hospital after she attempted to stab her mother with a knife. She had low grade fever, headaches and lethargy prior to presentation. Organic workup revealed serum and CSF anti-nmda receptor to be positive. She received early treatment with steroids and intravenous immunoglobulins and no longer harboured further homicidal ideations.

**Conclusions:**

Initial suspicion of anti-NDMA receptor encephalitis should be raised and early auto-immune workup performed in paediatric patients presenting with acute change in behavior especially in young females with no past medical or psychiatric history

**Disclosure:**

No significant relationships.

